# Auditory experience modulates fronto-parietal theta activity serving fluid intelligence

**DOI:** 10.1093/braincomms/fcac093

**Published:** 2022-04-05

**Authors:** Elizabeth Heinrichs-Graham, Elizabeth A. Walker, Brittany K. Taylor, Sophia C. Menting, Jacob A. Eastman, Michaela R. Frenzel, Ryan W. McCreery

**Affiliations:** 1 Cognitive and Sensory Imaging Laboratory, Institute for Human Neuroscience, Boys Town National Research Hospital, 14090 Mother Teresa Ln, Boys Town, NE 68010, USA; 2 Department of Pharmacology and Neuroscience, College of Medicine, Creighton University, Omaha, NE 68178, USA; 3 Center for Magnetoencephalography (MEG), University of Nebraska Medical Center (UNMC), Omaha, NE 68198, USA; 4 Wendell Johnson Speech and Hearing Center, Department of Communication Sciences and Disorders, University of Iowa, Iowa City, IA 52242, USA; 5 Department of Psychology, University of Nebraska - Lincoln, Lincoln, NE 68588, USA; 6 Audibility, Perception, and Cognition Laboratory, BTNRH, Omaha, NE 68010, USA

**Keywords:** audiology, hearing aids, neurophysiology, non-verbal cognition, oscillations

## Abstract

Children who are hard of hearing are at risk for developmental language and academic delays compared with children with normal hearing. Some work suggests that high-order cognitive function, including fluid intelligence, may relate to language and academic outcomes in children with hearing loss, but findings in these studies have been mixed and to date, there have been no studies of the whole-brain neural dynamics serving fluid intelligence in the context of hearing loss. To this end, this study sought to identify the impact of hearing loss and subsequent hearing aid use on the neural dynamics serving abstract reasoning in children who are hard of hearing relative to children with normal hearing using magnetoencephalography. We found significant elevations in occipital and parietal theta activity during early stimulus evaluation in children who are hard of hearing relative to normal-hearing peers. In addition, we found that greater hearing aid use was significantly related to reduced activity throughout the fronto-parietal network. Notably, there were no differences in alpha dynamics between groups during later-stage processing nor did alpha activity correlate with hearing aid use. These cross-sectional data suggest that differences in auditory experience lead to widespread alterations in the neural dynamics serving initial stimulus processing in fluid intelligence in children.

## Teaser

Heinrichs-Graham *et al*. report elevations in parieto-occipital theta activity during abstract reasoning in children with hearing loss relative to children with normal hearing. Theta activity throughout the fronto-parietal network was also related to the amount of hearing aid use, underscoring the importance of consistent hearing aid use on cognitive and brain development.

## Graphical Abstract

**Graphical abstract:**
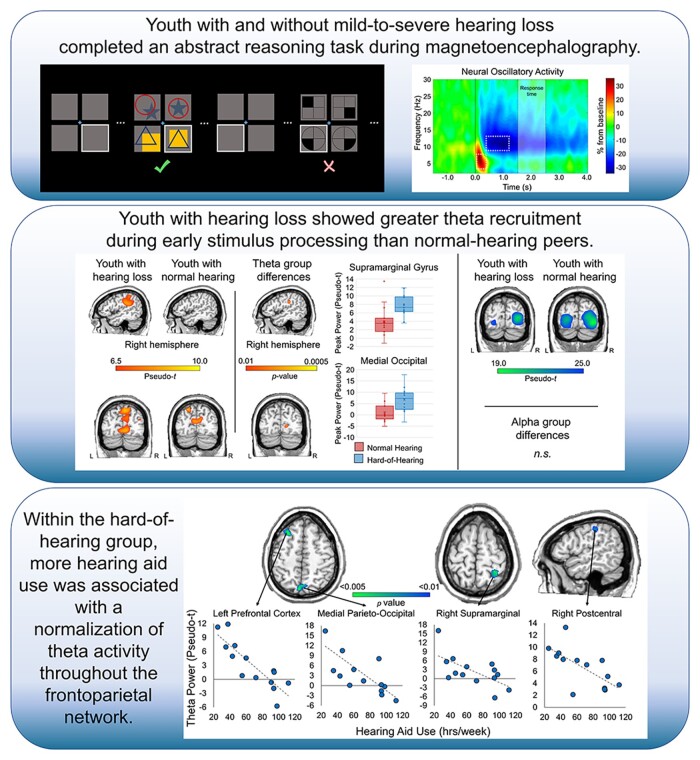

## Introduction

Abstract reasoning is a component of fluid intelligence that allows an individual to extrapolate a relationship between two or more objects or scenarios and applies this relational information to a new set of objects or scenarios. While fluid intelligence is a component of overall intelligence, the developmental trajectories of the components that make up overall intelligence (e.g. abstract reasoning, verbal comprehension, processing speed) can be dissociated. There is a consensus that abstract reasoning emerges between 2 and 3 years of age and develops rapidly from early childhood into adolescence before reaching maturity in early adulthood.^1^ Importantly, the developmental trajectory of fluid intelligence ability is significantly different than crystallized intelligence, supporting the notion that each is a unique subconstruct of generalized intelligence.^1^ Nonetheless, while fluid intelligence is dissociable from other types of cognition (e.g. working memory, attention, inhibition, language), it correlates with these processes^2–5^ and is a significant predictor of academic achievement among school-age children.^6–8^ Given its relationship with other cognitive abilities and academic outcomes in children, abstract reasoning is an important construct to consider in children who are at risk for developmental and academic delays.

One such developmental risk factor is congenital hearing loss. Children with bilateral hearing loss are at increased risk of developmental language and cognitive delays.^9–14^ This includes children who are hard of hearing (CHH; i.e. children with mild-to-severe hearing loss) as well as children who are deaf. Two recent large-scale studies of children and adolescents suggest that bilateral hearing loss leads to reduced performance on non-verbal intelligence tasks, including fluid intelligence.^15,16^ Most of the children in these studies had mild-to-moderate hearing loss, which suggests that even CHH are at risk of non-verbal cognitive delays compared to children with normal hearing (CNH). Nonetheless, these large-scale analyses did not take into account the presence of other comorbidities prevalent in children with hearing loss, so the impact of hearing loss on non-verbal intelligence over-and-above additional developmental disabilities remains unclear. Furthermore, we calculated effect sizes using the non-verbal intelligence data from these studies and found them to be relatively modest (e.g. Cohen’s *d* = 0.5),^16^ and thus smaller studies are less likely to have the sample sizes required to detect these subtle behavioural differences in non-verbal intelligence at the group level. Indeed, findings in other studies are mixed; some show specific non-verbal cognitive decrements (e.g. fluid intelligence, attention, executive function^17,18^), whereas others show no difference groupwise between CHH and CNH.^19^ However, numerous studies have shown that non-verbal intelligence predicts language ability in children with and without hearing loss, suggesting that fluid intelligence may moderate academic and language outcomes in this population.^20–24^ Because language issues pose the biggest risk to CHH, an understanding of how these cognitive processes are linked and affected by auditory experience is important for improving outcomes.

As alluded to above, the variability across studies between CHH and CNH and among CHH may be related to differences in auditory experience (i.e. a culmination of the basal level of hearing an individual has, as well as the quantity and quality of hearing intervention, if necessary) and thus, alterations in auditory experience may be able to explain differences in behaviour and neurophysiology between CHH and CNH on the group level, as well as the variability in outcomes within the hard-of-hearing population. CHH is increasingly identified with hearing loss via newborn hearing screening programmes and then fitted with hearing aids and enroled in early intervention within months of birth.^25^ However, 30–40% of early identified CHH experience persistent deficits in language,^9^ executive function^18^ and academic outcomes.^26^ Recent studies have found that CHH who have more consistent hearing aid use have better academic and language outcomes than CHH who have fewer hours of hearing aid use per day.^12,18,26^ Crucially, we recently reported that the amount of hearing aid use also significantly relates to neurophysiological markers of cognitive processing in CHH, such that greater hearing aid use (especially more than ∼8.5 h per day) served to significantly normalize patterns of neural activity during working memory processing in CHH.^27^ Nonetheless, the effects of individual differences in auditory experience on non-verbal intelligence have rarely been described in previous studies, and to date, there has been no investigation of the underlying neurophysiology.

Researchers have long attempted to determine the neural correlates of intelligence more broadly, as well as fluid intelligence as its own construct. The prevailing theory is the parietal-frontal intelligence theory (P-FIT).^28^ Jung and Haier^28^ performed a meta-analysis of 37 structural and functional neuroimaging studies of various intelligence constructs and found that grey matter volumes in the dorsolateral prefrontal cortex (DLPFC) and posterior parietal cortices, as well as temporal and occipital regions, reliably predicted intelligence and reasoning performance in humans. Importantly, longitudinal studies of development also showed that increases in grey matter in these regions in early childhood, as well as proportional decreases in cortical thickness in later adolescence (i.e. cortical thinning) positively correlated with performance on intelligence tests in children.^28^ These results have largely been corroborated in later studies of intelligence in children and adults.^29,30^ Most recently, Taylor *et al*.^31^ sought to identify the oscillatory neural dynamics that underlie abstract reasoning performance in children and adolescents. They found significant correlations between age and theta event-related synchronization (ERS) activity in the medial occipital and cerebellar cortices. They also found robust age-by-sex relationships throughout the fronto-parietal network, such that theta ERS activity in the dorsolateral lateral and superior prefrontal, posterior parietal, and occipital cortices increased as a function of age in males, whereas activity in these regions showed no change or a decrease as a function of age in females, suggesting differential maturation in these regions by sex.^31^ Nonetheless, the impact of auditory experience on these neural responses remains completely unknown. This is an important avenue, as it is possible that CHH show differential neural patterns despite having comparable performance, and this may give insight into the locus of variability in this population.

The present study sought to identify the impact of mild-to-severe hearing loss and subsequent hearing intervention on the neural dynamics serving abstract reasoning performance in children and adolescents. We recorded the neural oscillatory responses from a group of 7- to 15-year-old CHH and CNH using magnetoencephalography (MEG) while they performed an abstract reasoning task adapted from Raven’s Progressive Matrices. Given our sample size, we hypothesized that we would not detect significant decrements in abstract reasoning performance at the group level between CHH and CNH. However, we hypothesized that they would exhibit altered neural responses in fronto-parietal executive function regions during task performance. We also hypothesized that the amount of hearing aid use would significantly correlate with the neural responses serving abstract reasoning performance within CHH.

## Methods

### Participants

A total of 52 participants, including 27 CHH and 25 CNH, were initially consented from the local community to participate in this study. This sample size was selected based on our prior work showing differences in brain activity between CHH and CNH.^27,32^ All data collection occurred at the University of Nebraska Medical Center. Exclusionary criteria included any medical illness affecting CNS function, current or previous major neurological or psychiatric disorder, history of head trauma, current substance abuse, and/or the presence of irremovable ferromagnetic material in or on the body (e.g. dental braces, metal or battery-operated implants). After a complete description of the study was given to participants, written informed consent was obtained from the parent/guardian of the participant, and informed assent was obtained from the participant following the guidelines of the University of Nebraska Medical Center’s Institutional Review Board, which approved the study protocol. Exclusionary criteria were discovered in three CNH and four CHH after consent, and these seven participants were not included in the sample. Two additional participants (one CNH and one CHH) were not included in the analysis due to technical errors in MEG data acquisition, and one additional CHH chose to withdraw from the study before MEG recording. Finally, three CNH and five CHH were excluded due to an inability to perform the task (accuracy <50%; three CNH and four CHH) or irremovable movement artefacts during recording (one CHH). Thus, 19 CNH and 16 CHH were included in the analysis. The final sample demographics were as follows: CNH average age = 11.58 (SD = 1.97) years, range = 7.50–14.83 years, 8 females; CHH average age = 11.59 (SD = 2.02) years, range = 8.31–15.64 years, 6 females. There were no between-group differences in age, *t*(33) = 0.015, *P* = 0.988, or sex, *X*^2^ (1, *N* = 35) = 0.077, *P* = 0.782. Researchers were blinded to group membership during all preprocessing and processing of MEG and behavioural data until statistically necessary to avoid any potential bias.

### Neuropsychological and audiometric testing

All participants completed all four subtests of the Wechsler Abbreviated Scale of Intelligence (WASI-II^33^) to characterize their level of verbal and non-verbal cognitive function. The WASI-II consists of the following subtests: Vocabulary, Similarities, Block Design and Matrix Reasoning, which can be used to calculate an individual’s verbal, non-verbal and overall IQ. Scores on the Vocabulary and Similarities subtests are combined to create the Verbal Composite Index (VCI), which is a metric of verbal intelligence, whereas the Block Design and Matrix Reasoning scores are combined to create a Perceptual Reasoning Index (PRI), which is a measure of non-verbal intelligence. In addition, we calculated the degree of hearing loss [i.e. better ear pure-tone average (BEPTA)] for all CHH from the participants’ most recent clinical audiogram, which was completed within the past year and provided with parent consent. Audiograms consisted of air-conduction audiometric thresholds that had been measured at octave frequencies from 250 to 8000 Hz. The thresholds at 500, 1000, 2000 and 4000 Hz were averaged to calculate the pure-tone average (PTA) for each ear, and the PTA for the better ear was used to represent degree of hearing loss in the statistical analyses. Finally, for the CHH only, parents or guardians filled out questionnaires regarding their child’s hearing aid use during the school year, summer and weekends (e.g. ‘During the school year, how many hours a day does your child wear the aids Monday–Friday? Saturday–Sunday?’). We then calculated hearing aid use in a total number of hours per week, Monday through Sunday. For all analyses, we used the number of hours participants wore their hearing aids during the school year. The final sample of CHH had an average BEPTA of 42.80 dB (SD = 10.37 dB, range: 28.75–56.25 dB) and wore their hearing aids at an average of 70.50 h/week (SD = 26.48 h/week, range: 25–112 h/week).

### Experimental paradigm

**Figure 1:**
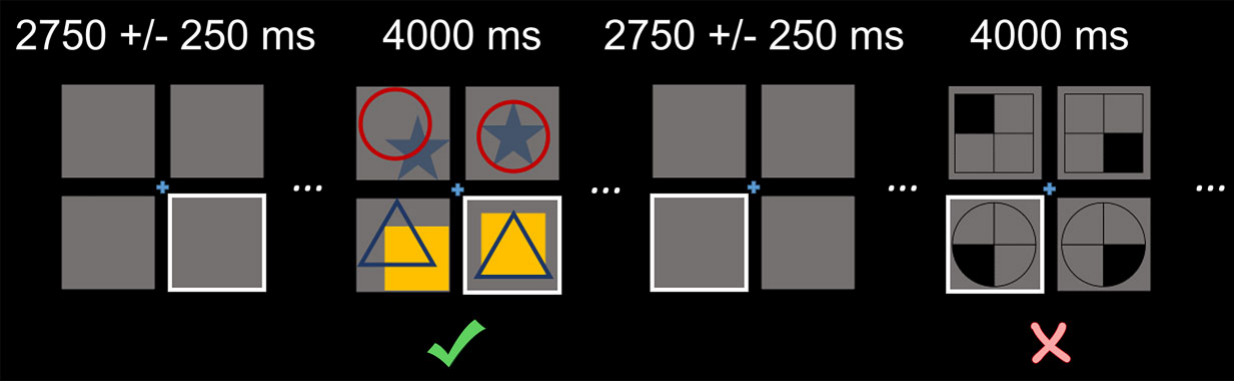
**Task paradigm.** Participants were initially presented with a fixation surrounded by a 4 × 4 square grid for 2.75 +/− 0.25 s, during which one of the bottom boxes was highlighted with a border. After this baseline period, the four squares were then populated with complex shapes for 4.0 s, during which participants were instructed to respond via button press whether the shapes in the highlighted box were related to the shapes in the other bottom box in the same way as the shapes in the top boxes. A total of 120 trials were presented.

During MEG recording, participants performed a non-progressive abstract reasoning task based on Raven’s Matrices. Participants were initially presented with a central crosshair that was surrounded by a blank 4-by-4 grid, with one of the bottom two blocks highlighted with a white box. After a 2.75 +/− 0.25 s baseline period, the four boxes were populated with complex shapes. The participant was instructed to determine the pattern between the shapes in the top two boxes and respond whether the shapes in the highlighted box completed the same pattern with the other bottom box as that found in the top two boxes. Patterns could be matched based on shape, colour, number or orientation and had either 0 or 1 relational item to consider, to maximize the likelihood that all participants could perform the task. Each trial lasted 6.5–7.0 s; Fig. 1 shows two example trials. Each participant completed 120 trials that were pseudorandomized based on whether the probe box completed the pattern or not. The task lasted ∼14 min, including a 30 s break in the middle.

### Magnetoencephalography data acquisition

Neuromagnetic data were sampled continuously at 1 kHz using a 306-channel Elekta/MEGIN Neuromag system with 306 sensors with an acquisition bandwidth of 0.1–330 Hz (Helsinki, Finland). All recordings were conducted in a one-layer magnetically shielded room with active shielding engaged. Before MEG measurement, four continuous head position indicator coils were attached to the subject’s head and localized, along with the three fiducial points and scalp surface, with a 3D digitizer (Fastrak 3SF0002, Polhemus Navigator Sciences, Colchester, VT, USA). Once the subject was positioned for MEG recording, an electric current with a unique frequency label (e.g. 322 Hz) was fed to each of the coils. This induced a measurable magnetic field and allowed each coil to be localized relative to the sensors throughout the recording session, which by proxy allowed the head position to be continuously monitored relative to the sensor array. Offline, MEG data from each subject were then individually corrected for head motion and subjected to noise reduction using the signal space separation method with a temporal extension ^34,35^. Head motion correction was such that the position of the head throughout the recording was aligned to the individual’s head position when the recording was initiated.

### Magnetoencephalography coregistration and structural MRI processing

Because head position indicator coil locations were also known in head coordinates, all MEG measurements could be transformed into a common coordinate system. With this coordinate system, each participant’s MEG data were coregistered with structural T_1_-weighted MRI data before source space analyses using Brain Electrical Source Analysis (BESA) MRI (Version 2.0). Structural MRI data were aligned parallel to the anterior and posterior commissures and transformed into the Talairach coordinate system.^36^ Following source analysis (i.e. beamforming), each subject’s functional images were transformed into standardized space using the transform applied to the structural MRI volume.

### Magnetoencephalography time–frequency transformation and statistics

Cardiac, eye blink and excessive eye movement artefacts were removed from the data using signal-space projection, which was accounted for during source reconstruction.^37^ The continuous magnetic time series was divided into epochs of 6.5 s duration, with the baseline being defined as −1.8 to −0.8 s before initial stimulus onset. Epochs containing artefacts were rejected based on a fixed threshold method, supplemented with a visual inspection. Artefact-free epochs were transformed into the time–frequency domain using complex demodulation^38^ (resolution: 1.0 Hz, 50 ms), and the resulting spectral power estimations per sensor were averaged over trials to generate time–frequency plots of mean spectral density. These sensor-level data were normalized by dividing the power value of each time–frequency bin by the respective bin’s baseline power, which was calculated as the mean power during the −1.8 to −0.8 s time period. This normalization allowed task-related power fluctuations to be visualized in sensor space.

The time–frequency windows subjected to beamforming (i.e. imaging) in this study were derived through a two-stage statistical analysis of the sensor-level spectrograms across the entire array of gradiometers (magnetometer data was not analyzed) during the 4 s stimulus presentation window, in line with our previous studies.^27,31,32,39^ Each data point in the spectrogram was initially evaluated using a mass univariate approach based on the general linear model. To reduce the risk of false-positive results while maintaining reasonable sensitivity, a two-stage procedure was followed to control for Type 1 error. In the first stage, one-sample *t*-tests were conducted on each data point, and the output spectrogram of *t*-values was thresholded at *P* < 0.05 to define time–frequency bins containing potentially significant oscillatory deviations across all participants. In Stage 2, time–frequency bins that survived the threshold were clustered with temporally and/or spectrally neighbouring bins that were also above the (*P* < 0.05) threshold on sensors within 4 cm of each other (i.e. spatial clustering), and a cluster value was derived by summing all of the *t*-values of all data points in the cluster. Non-parametric permutation testing was then used to derive a distribution of cluster values, and the significance level of the observed clusters (from Stage 1) was tested directly using this distribution^40,41^. For each comparison, at least 10 000 permutations were computed to build a distribution of cluster values. Based on these analyses, the time–frequency windows that contained significant oscillatory events across all participants during the encoding and maintenance phases were subjected to beamforming.

### Magnetoencephalography source imaging and statistical analysis

Cortical networks were imaged through an extension of the linearly constrained minimum variance vector beamformer, which employs spatial filters in the frequency domain to calculate source power for the entire brain volume.^42–44^ The single images are derived from the cross-spectral densities of all combinations of MEG gradiometers averaged over the time–frequency range of interest and the solution of the forward problem for each location on a grid specified by input voxel space. Following convention, we computed noise-normalized, differential source power per voxel in each participant using active (i.e. task) and passive (i.e. baseline) periods of equal duration and bandwidth.^44^ Such images are typically referred to as pseudo-*t* maps, with units (i.e. pseudo-*t*) that reflect noise-normalized power differences per voxel. Magnetoencephalography preprocessing and imaging used the BESA (version 6.0) software.

Normalized differential source power was computed for the selected time–frequency bands, using a baseline of equal length, over the entire brain volume per participant at 4.0 × 4.0 × 4.0 mm resolution. Each participant’s functional images were then transformed into standardized space using the transform that was previously applied to the structural images. Then, whole-brain independent samples *t*-tests were performed to dissociate the impact of hearing loss on the neural dynamics serving abstract reasoning. In addition, whole-brain Pearson correlations were performed to dissociate the impact of hearing aid use on abstract reasoning-related neural activity. We controlled for the degree of hearing loss in all hearing aid use analyses. All output statistical maps were adjusted for multiple comparisons using a spatial extent threshold (i.e. cluster restriction; *k* = 6 contiguous 4 mm^3^ voxels) based on the theory of Gaussian random fields.^45–47^ Basically, statistical maps were initially thresholded at *P* < 0.005 and then a cluster-based correction method was applied such that at least six contiguous voxels must be significant at *P* < 0.005 in order for a cluster to be considered significant.

### Data availability

The data that support the findings of this study are available from the corresponding author, upon reasonable request.

## Results

**Figure 2:**
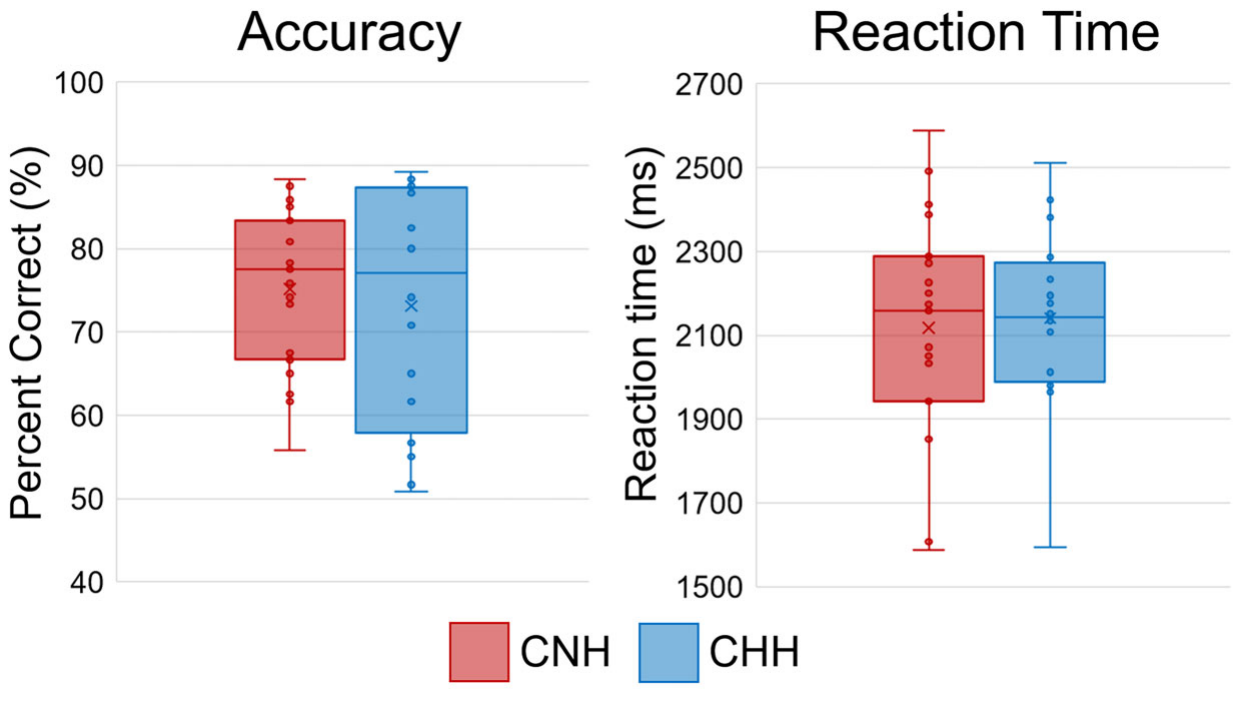
**Behavioural results.** Box plots of accuracy (in percent correct; left) and reaction time (in ms; right) are shown. The centre line within each box denotes the median frequency, and the *bottom* and *top* of each box designate the interquartile data. Lower and upper stems reflect the minimum and maximum values. CNH (*N* = 19) are shown in on the left of each graph, whereas children who are hard of hearing (CHH; *N* = 16) are shown on the right of each graph. There were no significant group differences in accuracy, *t*(25.4) = 0.478, *P* = .636, or reaction time *t*(33) = −0.276, *P* = 0.785. However, CHH showed significantly higher variability in accuracy than CNH.

Accuracy data did not pass Levene’s test for equality of variances (CHH had significantly more variability than CNH), so we report the results of that *t*-test with equal variance not assumed. Behaviourally, there were no differences in task performance between groups, accuracy: *t*(25.4) = 0.478, *P* = 0.636, CNH: 75.1% (SD = 9.6%), CHH: 73.1% (SD = 14.3%); reaction time: *t*(33) = −0.276, *P* = .785, CNH: 2117 ms (SD = 271 ms), CHH: 2141 ms (SD = 217 ms). Figure 2 shows the behavioural results. As expected, accuracy on the task correlated significantly with the Matrix Reasoning subtest of the WASI, as well as PRI scores more broadly [*r*(35) = 0.443, *P* = 0.008 and *r*(35) = 0.439, *P* = 0.009, respectively)] suggesting that this task likely tapped the same cognitive constructs as these well-studied abstract reasoning and non-verbal intelligence metrics. Both correct and incorrect trials were subject to preprocessing. Following artefact rejection, an average of 99.79 (SD = 8.92) trials for CNH and 103.44 (SD = 4.47) trials for CHH were included in analysis; this difference was not significant, *t*(33) = 1.485, *P* = 0.147.

### Time–frequency dynamics serving abstract reasoning

**Figure 3:**
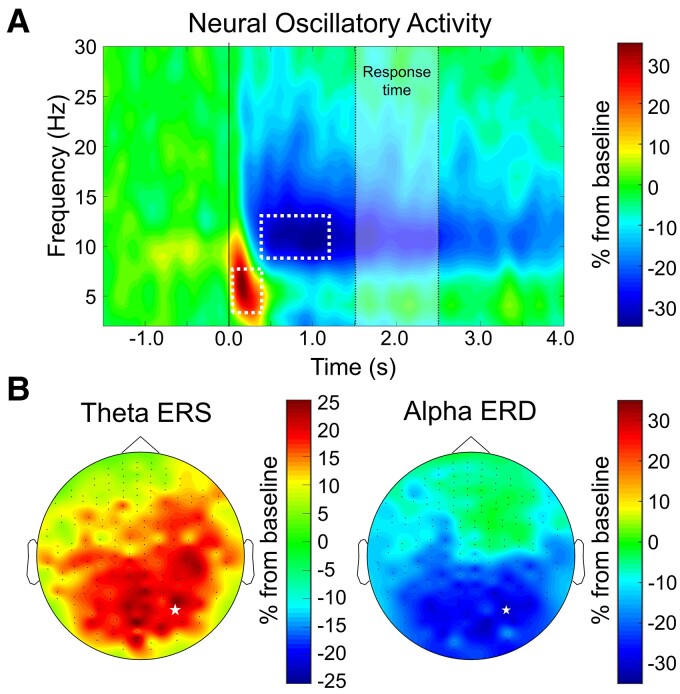
**Significant time–frequency windows during abstract reasoning.** (**A**) Time–frequency spectrogram, denoting the spectral power change as a percentage (+/–) from baseline as a function of time, from a representative sensor averaged across CNH and CHH, with time (in s; 0.0 s = stimulus onset) on the *x* axis and frequency (in Hz) on the *y* axis. Dotted boxes denote components selected for source imaging. (**B**) Topographic distribution of activity across all sensors within each time–frequency window (left: theta ERS from 3–7 Hz, 50–400 ms); right: alpha ERD from 8–13 Hz, 400–1200 ms). Note that the same sensor is highlighted with a white star in each topographic map and shown in the top panel.

Time–frequency bins of oscillatory activity to be imaged were identified across both groups. The two-stage statistical analysis of the sensor-level time–frequency spectrograms resulted in two significant time–frequency bins. There was a significant theta (3–7 Hz) ERS that was identified in a large number of posterior and medial sensors from 50 to 400 ms after stimulus onset (*P* < 0.0001, corrected). This response was followed by a significant sustained alpha (8–13 Hz) event-related desynchronization (ERD) in a large number of posterior sensors that was sustained until after the average response onset (*P* < 0.0001, corrected). Because we sought to determine the impact of hearing loss on abstract reasoning processing, we focused our analysis of alpha dynamics to the time period before the earliest response onset (i.e. before 1200 ms). Figure. 3 shows a time–frequency spectrogram from a representative sensor, as well as the sensor-level topography of each response to be imaged. These time–frequency bins (theta ERS: 3–7 Hz, 50–400 ms; alpha ERD: 8–13 Hz, 400–1200 ms) were independently imaged in each participant to determine the neural loci of these responses.

### Group differences in theta but not alpha activity during abstract reasoning

**Figure 4:**
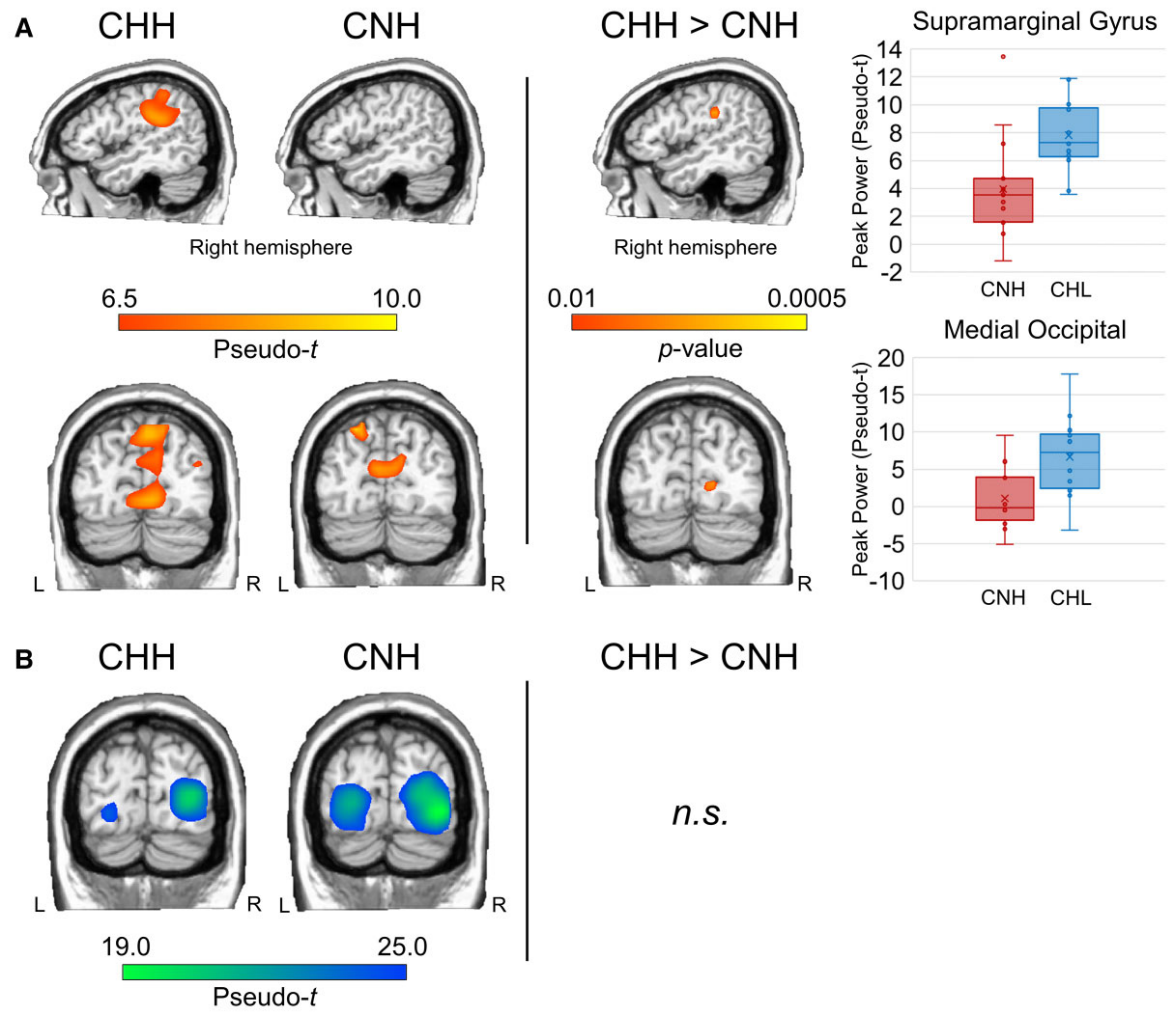
**Group-level differences in neural dynamics serving abstract reasoning.** (**A**) Left: Grand averages (pseudo-*t*) of theta ERS activity for CHH (left) and CNH (right). Right hemispheric activity is shown on the top, while occipital activity is shown on the bottom. Middle: Whole-brain statistical maps between CHH and CNH are shown. Right hemispheric activity is shown on the top, while occipital activity is shown on the bottom. Images are thresholded at *P* < 0.01 to aid in visualization. Right: Box-and-whisker plots of peak activity (pseudo-t) in these regions are shown on the right (CNH: left, CHH: right). (**B**) Left: Grand averages (pseudo-t) of alpha ERD activity for CHH (left) and CNH (right). Right: Whole-brain statistical comparisons showed no significant differences in alpha ERD activity between groups.

Group-averaged whole-brain maps of each response show differential activation of occipital and parietal regions in each group (see Figs. 4A and B, left). As described previously, whole-brain independent sample *t*-tests (CHH vs. CNH) were performed on the theta ERS and alpha ERD images separately. Resultant images were thresholded at *P* < 0.005 and corrected for multiple comparisons (*k* = 6, 4 mm^3^ voxels). We found significant differences in theta ERS activity between CNH and CHH in the medial occipital cortex, as well as the right supramarginal gyrus; CHH showed stronger ERS responses than CNH in both regions (*P* < 0.005, corrected; Fig. 4A, right). We found no significant differences in alpha ERD activity between groups.

### Impact of hearing aid use on neural dynamics serving abstract reasoning

Given the increasing evidence that consistent hearing aid use moderates language, executive function, and academic outcomes in CHH^12,18,26^ and may have cascading effects on cognitive neurophysiology,^27^ we next sought to determine whether the number of hours that CHH wore their hearing aids also modified their neural dynamics underlying abstract reasoning. To this end, the total number of hours of hearing aid use (Monday–Sunday, during the school year) were entered into a voxel-wise whole-brain correlation analysis with theta ERS and alpha ERD maps separately, with BEPTA acting as a covariate. As described previously, the resultant correlation maps were thresholded at *P* < 0.005 and corrected for multiple comparisons using a cluster threshold of *k* = 6 contiguous 4 mm^3^ voxels.

**Figure 5:**
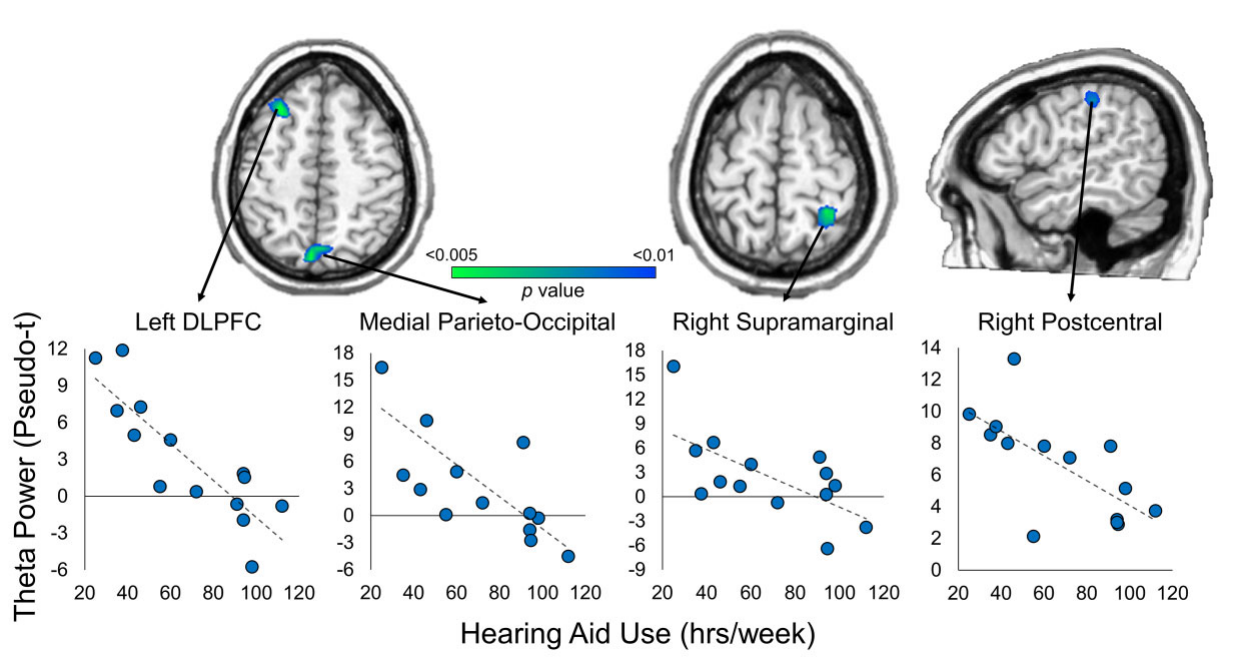
**Whole-brain correlation between theta ERS activity and hearing aid use.** Degree of hearing loss (in dB) acted as a covariate. Images are thresholded at *P* < 0.01 to aid in visualization; however, all results were thresholded at *P* < 0.005, corrected. Scatterplots denote total hours of hearing aid use (hours/week) on the *x* axis and peak activity values (pseudo-*t*) on the *y* axis. There were significant negative correlations between hearing aid use and activity in the left DLPFC, medial occipital cortex, right supramarginal gyrus and right postcentral gyrus (*P* < 0.005, corrected).

Significant negative correlations between theta ERS activity and hearing aid use were found throughout the fronto-parietal network (*P* < 0.005, corrected). Specifically, there were significant negative correlations in the left DLPFC; (peak *r*^2^ = 0.640), medial parieto-occipital cortex (peak *r*^2^ = 0.629) and right supramarginal gyrus (peak *r*^2^ = 0.682). There was also a significant negative correlation between hearing aid use and theta activity in the right postcentral gyrus (peak *r*^2^ = 0.686). In all of these regions, there was less theta activity with more hearing aid use (Fig. 5), controlling for a degree of hearing loss. There were no significant correlations found between hearing aid use and alpha ERD activity, controlling for the degree of hearing loss.

## Discussion

In this study, we sought to identify the impact of mild-to-severe hearing loss and subsequent amount of hearing aid use on the neural responses underlying abstract reasoning performance. We found that despite comparable behavioural performance between groups, theta ERS activity during early stimulus processing was significantly elevated in CHH relative to CNH in the right supramarginal gyrus and medial occipital cortex. In contrast, alpha ERD activity during later stages of stimulus processing was not significantly different between groups. Within the CHH, theta activity in the fronto-parietal network including the left DLPFC, medial parieto-occipital cortex, and right supramarginal gyrus, as well as the right postcentral gyrus, were significantly correlated with hearing aid use after controlling for the degree of hearing loss in CHH, such that CHH who wore their hearing aids more consistently recruited these regions less. Alpha ERD activity during this task did not correlate with hearing aid use. Below we discuss the implications of these results in understanding the relationship between hearing loss and non-verbal cognitive development in children.

CHH showed increased theta ERS activity relative to CNH in the medial occipital cortex along the calcarine fissure during early stimulus processing. This increased theta ERS activity is likely indicative of a heightened response during initial bottom-up visual processing, as the calcarine fissure hosts the primary visual cortex. This visual theta response has been shown to be enhanced with increased attention to a visual stimulus.^48^ Thus, CHH may have been using greater attentional resources than CNH during the initial processing of the complex stimulus presented in this task. It is possible that this increased attention reflects a greater need or it could indicate a more general mechanism by which CHH process their environment due to degraded auditory input. In other words, because CHH likely does not depend on auditory attention cues to the same level as CNH, they potentially have heightened visual attention mechanisms in place to compensate. For example, Jerger *et al.*^49^ identified whether changes in language processing were due to differences in basic visual or auditory processing using a speech perception task with auditory, visual (static or dynamic), or audiovisual stimuli. The authors showed that overall, if the speech is accompanied by a visual cue (i.e. either a static face or a face uttering the speech sound), then both CNH and CHH responded more quickly to the stimulus. However, CHH responded more quickly when the visual cue was present relative to auditory-only, both when the face was dynamic (i.e. uttering the speech sound) and when it was static, while CNH only responded more quickly than auditory-only if the visual cue was dynamic.^49^ This suggests that CHH utilize greater visual attention resources overall to understand speech sounds. Extending this to the current study, it is possible that greater attention to visual input is a domain-general mechanism that applies not only to speech perception but also attention more broadly in CHH. Future studies should investigate the role of visual neural activity in moderating behavioural outcomes in CHH, especially using multimodal methods such as neuroimaging with simultaneous eye-tracking.

In addition to elevated theta ERS activity in the primary visual cortices, CHH showed increased theta ERS activity in the supramarginal gyrus relative to CNH. While the supramarginal gyrus is known to be associated with a variety of high-order cognitive tasks including expressive language function,^50^ emotion processing^51^ and spatial awareness,^52^ recent work suggests that the supramarginal gyrus and lateral inferior parietal cortex are also crucial to object categorization and perception–action sequences,^53^ as well as visual attention.^54^ Thus, greater theta activity in these regions during the early stages of processing in CHH relative to CNH may be related to visual processing and categorization of the stimuli, as is necessary in this task before making a decision regarding the pattern compliance of the target. It is possible that this increased activity is compensatory, such that there may be decreased efficiency in the neural network and/or an increased allocation of the neural resources serving pattern recognition and visual attention, concurrent with the increased visual attention resources found in occipital regions as described previously. Taken together, the results of the present study suggest that, at least at the group level, CHH utilizes increased neural resources during the initial stages of visual processing and pattern recognition than CNH. The lack of difference in later-stage alpha ERD dynamics or overall task behaviour between CHH and CNH suggests that this increased initial theta activity serves as a successful compensatory mechanism with which CHH can adequately perform the task at levels similar to CNH.

Perhaps, our most striking pattern of results was found *within* the CHH. We found a significant modulation of theta activity throughout the fronto-parietal executive function network with hearing aid use within the CHH, such that CHH who wore their hearing aids more consistently had less activity in the fronto-parietal network, while those who did not wear their hearing aids consistently had elevated activity throughout this network. As described previously, P-FIT suggests that the structure and function of the bilateral DLPFC, posterior parietal cortices and occipital cortices are causally related to fluid intelligence in children and adults.^28–30^ Thus, an increase in activity in this network with decreased hearing aid use could reflect the greater allocation of neuronal resources to perform the task (i.e. less efficiency within the network). This interpretation has widespread implication for real-life behaviour in CHH. For example, it is possible that CHH performed at or near levels of CNH on this task at the group level in a controlled, decontextualized laboratory setting, but the neural systems serving these processes were taxed to a greater extent in CHH who did not wear their hearing aids consistently. If this is the case, real-world settings in which there are distractions and/or the need to multitask could result in an overburden of the neural networks serving these cognitive processes in CHH and may lead to subsequent behavioural decrements. This proposed dose–response relationship (i.e. the amount of neural activity required for an individual to perform a task relative to the task demands) may explain the variability in previous behavioural findings across the literature and underscores the importance of consistent hearing aid use for brain and behavioural health in CHH. In sum, these data are the first to suggest that hearing aid use is directly related to the neural dynamics serving non-verbal executive function, above and beyond any effects of the degree of hearing loss, and suggests that neuromaging may be helpful in quantifying the impact of therapeutic intervention parameters on cognitive and neural development in CHH. In addition, hearing aid use is not the only audiological metric of importance in the context of hearing intervention. Future studies should investigate the added impacts of hearing aid fit (e.g. audibility) and age of intervention.

In conclusion, this study is the first to determine the impact of hearing loss and subsequent hearing aid use on the spatiotemporal dynamics underlying abstract reasoning in children. We found that CHH showed increased theta activity in the right supramarginal gyrus and medial occipital regions during the early stages of stimulus processing, but no significant changes in neural activity during later stages of the task. Thus, it is possible that any behavioural differences in fluid intelligence found in other studies are due to the differences in the initial processing of the stimuli, which may also have cascading cumulative effects on later-stage cognitive demands (e.g. applying patterns or rules to the stimuli in question, decision-making). In addition, we found significant correlations between hearing aid use and early theta activity throughout the fronto-parietal network, such that there was decreased recruitment of these regions with increased hearing aid use in CHH. This pattern of results suggests dissociable impacts of hearing loss and hearing aid use on the oscillatory dynamics serving fluid intelligence and that CHH may tax their neural systems to a greater extent than CNH to perform at the same behavioural level. Finally, increased recruitment of neural resources, especially in the fronto-parietal executive function network, is exacerbated in CHH who do not wear their hearing aids consistently. More broadly, these data underscore the importance of looking at both groupwise comparisons and individual differences in the context of CHH. Future studies should enroll a larger, longitudinal cohort of CHH and CNH to investigate the developmental trajectory of performance and related brain dynamics with regard to non-verbal intelligence.

## Abbreviations

BEPTA =: better ear pure-tone average
BESA =: brain electrical source analysis
CHH =: children who are hard of hearing
CNH =: children with normal hearing
DLPFC =: dorsolateral prefrontal cortex
ERD =: event-related desynchronization
ERS =: event-related synchronization
MEG =: magnetoencephalography
P-FIT =: parieto-frontal intelligence theory
PRI =: perceptual reasoning index
PTA =: pure-tone average
VCI =: verbal composite index
WASI =: Wechsler Abbreviated Scale of Intelligence
